# Long-Term 5-HT_1A_ Receptor Agonist NLX-112 Treatment Improves Functional Recovery After Spinal Cord Injury

**DOI:** 10.3390/ijms26010239

**Published:** 2024-12-30

**Authors:** Ching-Yi Lin, Kevin Li, Thomas Gitchell, Yu-Shang Lee

**Affiliations:** 1 Department of Neurosciences, Lerner Research Institute, Cleveland Clinic, Cleveland, OH 44195, USA; 2 Department of Medicine, Cleveland Clinic Lerner College of Medicine, Case Western Reserve University, Cleveland, OH 44195, USA

**Keywords:** spinal cord injury (SCI), serotonin, serotonergic system, 5-HT_1A_ receptor, 5-HT_1A_ receptor agonist, NLX-112, locomotion, lower urinary tract function

## Abstract

Spinal cord injury (SCI) results in functional deficits below the injured spinal level. The descending serotonergic system in the spinal cord is critically involved in the control of motor and autonomic functions. Specifically, SCI damages the projections of serotonergic fibers, which leads to reduced serotonin inputs and increased amounts of spinal serotonergic receptors. Our previous pharmacological study demonstrated that brief administration of a highly selective 5-HT_1A_ receptor agonist, NLX-112, improves lower urinary tract (LUT) function at the termination stage of thoracic 8 (T8) contusive SCI in rats. However, whether chronic activation of serotonin 5-HT_1A_ receptors by NLX-112 after SCI is beneficial remains an unanswered question. Here, we evaluated the efficacy of long-term NLX-112 intervention starting from two weeks post-T8 contusive SCI for an additional six weeks. We evaluated locomotion, LUT function, bladder morphology, and the number of spinal 5-HT_1A_ receptors in both L4 and L6/S1 spinal cord segments. Our results indicate that NLX-112 treatment significantly improves locomotion in a dose-dependent fashion, improves LUT function, reduces bladder weight and bladder wall thickness, and reduces the SCI-upregulated spinal 5-HT_1A_ receptors compared to vehicle-treated SCI animals. These data suggest promising therapeutic potential for long-term NLX-112 activation of 5-HT_1A_ receptors to treat SCI.

## 1. Introduction

Spinal cord injury (SCI) above the lumbosacral level results in lower urinary tract dysfunction, including (1) detrusor hyperreflexia, wherein bladder compliance is low, and (2) a lack of external urethral sphincter (EUS) control, leading to detrusor sphincter dyssynergia (DSD) with poor voiding efficiency. These dysfunctions are ranked highly by SCI patients in terms of the level of significant impact on their overall quality of life [1,2,3]. Therefore, the need to discover effective interventions to improve/restore bladder function is urgent.

Experimental studies in animals have shown dense innervation of serotonergic (5-HT) fibers and multiple 5-HT receptors in the spinal reflex circuits that control the voiding function. Particularly, pharmacological activation of 5-HT_1A_ receptors after SCI resulted in improved EUS activity in rats [4,5,6] and inhibited detrusor activity in cats [7]. Thus, 5-HT_1A_ receptor agonists become attractive potential therapeutic agents to treat neurogenic bladder after SCI. However, the 5-HT_1A_ receptor agonists that are currently available have substantial limitations. For example, the prototypical ligand, 8-hydroxy-2-(di-n-propylamino)tetralin (8-OH-DPAT) activates both 5-HT_1A_ and 5-HT_7_ receptors. Clinical compounds such as buspirone have poor selectivity for, and only partial agonist activity at, 5-HT_1A_ receptors [8]. To circumvent these limitations, one advanced compound, NLX-112 (a.k.a. Befiradol or F13640), has been developed as a very potent, highly selective and fully efficacious 5-HT_1A_ agonist with the potential to be further advanced as a novel therapeutic agent. NLX-112 is being tested in a Phase II clinical study for the treatment of L-DOPA-induced dyskinesia in Parkinson’s disease patients. In addition, NLX-112 has shown beneficial effects for alleviating pain response after SCI [9,10], which is a similar effect as that elicited by 8-OH-DPAT treatment [11].

Although activation of 5-HT receptors using 8-OH-DPAT or quipazine produced beneficial effects on motor function in previous studies [12,13,14], the ability of long-term activation of 5-HT_1A_ receptors to improve lower urinary tract (LUT) function remains unanswered. In addition, both 8-OH-DPAT and quipazine are experimental drugs that cannot be used for clinical application. Therefore, NLX-112 could potentially be a feasible intervention for translational purposes. In our previous study [15], we demonstrated that acute pharmacological activation of 5-HT_1A_ receptors by NLX-112 through an intravenous injection can reduce bladder hyperactivity, improve voiding efficiency, and improve EUS activity in rats with both T8 complete SCI and T8 contusive SCI [15]. In the present study, we determined the therapeutic efficacy of long-term NLX-112 treatment beginning 2 weeks after injury by evaluating both anatomical and behavioral outcomes in adult rats with T8 contusive SCI. The expression of spinal 5-HT_1A_ receptors caudal to the injured site by immunohistochemistry (IHC) was investigated. Locomotor function was assessed by open field locomotion test using the Basso, Beattie, and Bresnahan (BBB) locomotor rating scale. LUT function was determined by a comprehensive awake cystometrogram (CMG) and EUS electromyogram (EMG) recording, along with a detrusor morphology study.

## 2. Results

### 2.1. Two-Week Delayed NLX-112 Treatment Significantly Improves Locomotion After SCI in a Dose-Dependent Manner

**Figure 1:**
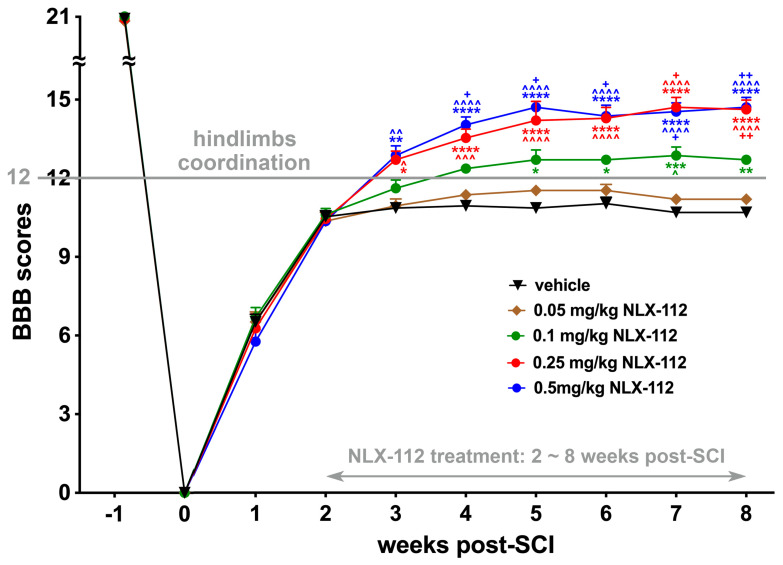
Locomotor recovery after contusive SCI was assessed using the BBB open-field test. NLX-112 (5-HT_1A_ receptor agonist) treatments were started at 2 weeks post-SCI and lasted for an additional 6 weeks. Statistical analyses of BBB scores (mean ± SEM) showed that NLX-112 treatments significantly increased BBB scores over the observation period in a dose-dependent fashion. * *p* < 0.05, ** *p* < 0.01, *** *p* < 0.001, and **** *p* < 0.0001 vs. SCI + vehicle animals; ^ *p* < 0.05, ^^ *p* < 0.01, ^^^ *p* < 0.001, and ^^^^ *p* < 0.0001 vs. SCI + 0.05 mg/kg NLX-112 animals; + *p* < 0.05, and ++ *p* < 0.01 vs. SCI + 0.1 mg/kg NLX-112 animals (two-way ANOVA with a Tukey’s post hoc test). N = 6 per group.

We first investigated the effect of long-term NLX-112 activation of 5-HT_1A_ receptors on hindlimb locomotion after T8 contusive SCI. The BBB open-field locomotion test was used to assess hindlimb locomotion after the SCI procedure and after NLX-112 or vehicle treatment until the end of the observation period among the SCI groups with different doses of NLX-112 (Figure 1). At two weeks post-SCI before treatment began, the majority of SCI animals showed BBB scores of 10–11, indicating weight-support stepping without hindlimb coordination. Following an additional 6 weeks of treatment, vehicle-treated SCI animals and animals administered the lowest dose (0.05 mg/kg) of NLX-112 showed only slightly improved BBB scores at the end of observation (i.e., 8 weeks post-SCI) to an average of 10.67 ± 0.142 and 11.17 ± 0.207 (mean ± SEM), respectively. However, there was no significant difference in locomotion improvement between vehicle-treated and 0.05 mg/kg NLX-112-treated SCI animals. The group administered the next highest dose (0.1 mg/kg) of NLX-112 treatment started to show significant improvement to a BBB score of 12.67 ± 0.142 at the end of the study, as compared to vehicle-treated or 0.05 mg/kg NLX-112-treated animals. At the higher NLX-112 doses of 0.25 mg/kg and 0.5 mg/kg, there were further significant improvements in BBB scores at the end of observation to an average of 14.58 ± 0.358 and 14.67 ± 0.376, respectively, when compared to what was observed with 0.1 mg/kg NLX-112 treatment. However, there was no significant difference between 0.25 mg/kg-treated (14.58 ± 0.358 BBB score) and 0.5 mg/kg-treated animals (14.67 ± 0.376 BBB score) at the end of the study. Therefore, the dose of 0.25 mg/kg was established as the optimal dose of NLX-112 for the subsequent long-term application experiments.

### 2.2. NLX-112 Treatment Significantly Improves LUT Function at 8 Weeks After SCI

**Figure 2:**
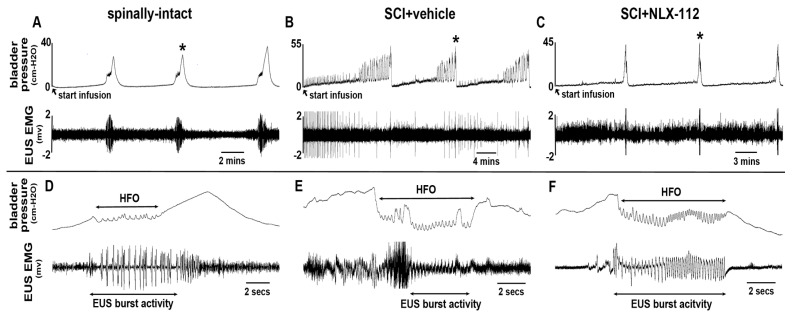
The LUT function of SCI animals treated with NLX-112 was analyzed by CMG and EUS EMG recordings. Three representative consecutive voiding cycles with CMG and EUS EMG recordings are shown from the (**A**) spinally-intact, (**B**) SCI + vehicle, and (**C**) SCI + NLX-112 groups. Note that the SCI + vehicle group needed less time to reach the second voiding contraction after the first voiding contraction, which had low voiding efficiency. Highlights of the representative HFO and EUS EMG activity during the micturition point (asterisks in **A**–**C**) are shown in (**D**–**F**). Note that there were relatively clear and consistent bursting activities in NLX-112-treated animals compared to SCI + vehicle-treated animals.

Awake CMG and EUS EMG recording at the end of the observation period was used to assess LUT function, including both detrusor and EUS activity, among all animal groups. In spinally-intact animals with continuous saline infusion, there was no detrusor contraction in the CMG recording during the bladder-filling phase until the micturition point (Figure 2A,D). The EUS EMG showed tonic activity in the bladder-filling phase. During the voiding period, high-frequency oscillation (HFO) detrusor activity was associated with the burst activity of EUS, which released fluid from the bladder (Figure 2A,D). The patterns of detrusor and EUS activities were consistent in the repetitive voiding cycles in spinally-intact animals. In the SCI + vehicle animals, the CMG recordings indicated the typical neurogenic bladder with detrusor hyperactivity associated with many non-voiding contractions during the bladder-filling phase (Figure 2B). During the voiding period, the detrusor HFO remained, but poor EUS bursting or mixing with tonic activities was observed at the same time (Figure 2E). In the SCI + NLX-112 (0.25 mg/kg)-treated animals, overall improvements in both CMG and EUS EMG recordings were observed compared to the SCI + vehicle-treated animals. LUT function in the NLX-112-treated animals appeared closer to the spinally-intact animals. Particularly, there was a significant reduction of detrusor hyperactivity during the filling phase (Figure 2C) and improvements in EUS bursting activity during the micturition period (Figure 2F) as compared to the SCI + vehicle-treated animals.

**Figure 3:**
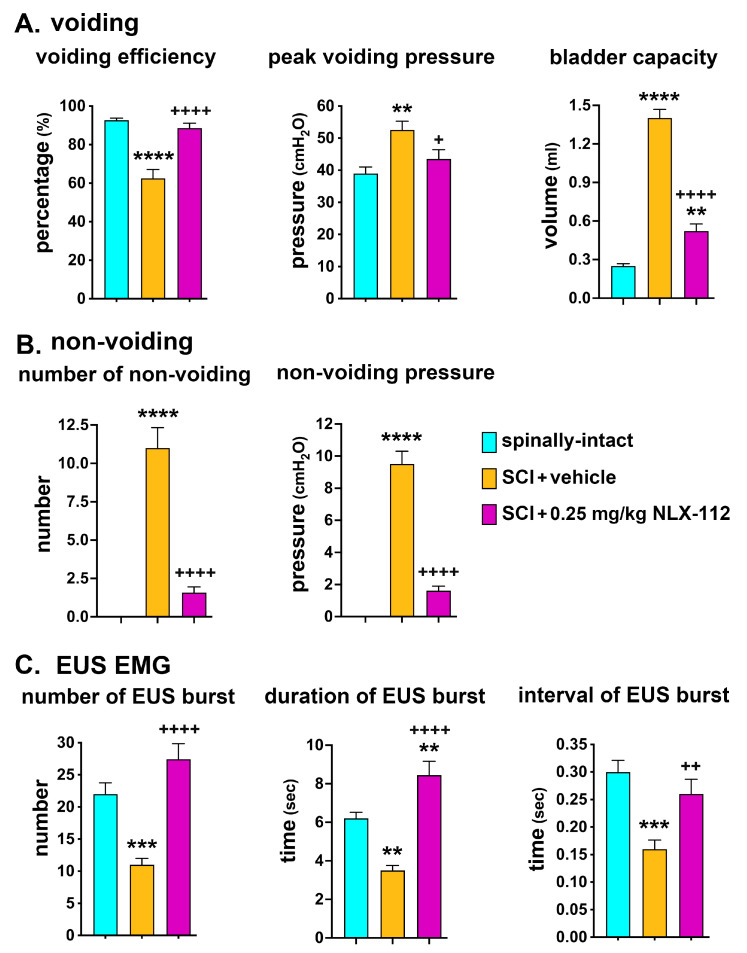
Effects of long-term systemic delivery of NLX-112 on micturition reflexes in T8 contused animals. Statistical analyses of parameters (mean ± SEM) representing voiding (**A**), non-voiding (**B**), and EUS EMG (**C**) activity recorded in the spinally-intact, SCI + vehicle, and SCI + NLX-112 groups. ** *p* < 0.01, *** *p* < 0.001, and **** *p* < 0.0001 vs. the spinally intact group; + *p* < 0.05, ++ *p* < 0.01, and ++++ *p* < 0.0001 vs. the SCI + vehicle group (one-way ANOVA with a Tukey’s post hoc test). N = 6 or 8 per group.

Parameters used to analyze the CMG recordings included voiding (voiding efficiency, peak voiding pressure, and bladder capacity) and non-voiding (number of non-voiding and non-voiding pressure). For EUS EMG, the number, duration, and interval of EUS burst activity were analyzed. In the voiding parameters, the SCI + vehicle animals showed significant decreases in voiding efficiency and significant increases in both peak voiding pressure and bladder capacity compared to the spinally-intact animals (Figure 3A). No non-voiding contractions were observed in the spinally-intact animals (Figure 3B). The SCI + vehicle group showed significant increases in both the number and amplitude of non-voiding contractions when compared to the spinally-intact animals (Figure 3B). With NLX-112 treatment, the SCI animals showed significant increases in voiding efficiency but significant decreases in peak voiding pressure, bladder capacity, and the number and amplitude of non-voiding contractions as compared to the SCI + vehicle-treated animals (Figure 3A,B). In the EUS EMG analysis, the SCI + vehicle animals showed significant decreases in the number, duration, and interval of EUS burst activity during micturition compared to the spinally-intact animals (Figure 3C). With NLX-112 treatment, EUS activity was significantly improved by increases in number, duration, and interval, which in turn led to better voiding efficiency following SCI (Figure 3C). The NLX-112-treated animals also had higher numbers and durations of EUS bursting activity than spinally-intact animals, which may be due to the extra work required to empty more urine volume because of the bladder size being increased after SCI.

### 2.3. NLX-112 Treatment Significantly Improves Bladder Morphology After SCI

**Figure 4:**
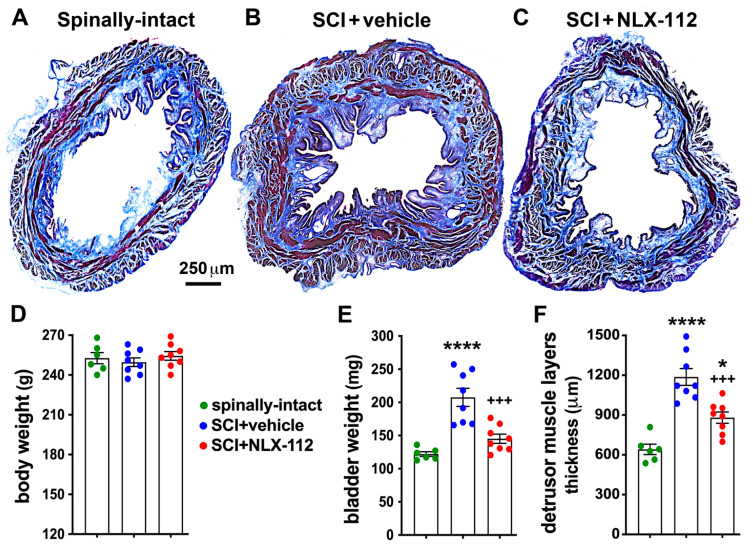
NLX-112 treatment significantly improved bladder morphology after SCI. Representative transverse bladder sections stained with Masson’s trichrome are shown for spinally-intact (**A**), SCI + vehicle (**B**), and SCI + NLX-112 (**C**) animals. The red color indicates muscle fibers and the blue color indicates collagen. Statistical analyses of animal body weight (**D**), bladder weight (**E**), and the thickness of detrusor muscle layers (**F**) are shown as mean ± SEM for the three animal groups. * *p* < 0.05, and **** *p* < 0.0001 vs. spinally-intact group; +++ *p* < 0.001 vs. SCI + vehicle group (one-way ANOVA with a Tukey’s post hoc test). N = 6 or 8 per group.

SCI-induced detrusor overactivity can lead to increased bladder size and weight. We thus further investigated whether NLX-112 treatment could alter bladder morphology after SCI. We assessed bladder morphology by measuring bladder weight and the thickness of the detrusor. There were no significant differences in body weight among the three groups (Figure 4D). The SCI + vehicle group showed significant increases in bladder weight and detrusor thickness compared to the spinally-intact animals (Figure 4A,B,E,F). The SCI + NLX-112 group showed slight but not significant increases in bladder weight but significant increases in detrusor thickness compared to the spinally-intact group (Figure 4A,C,E,F). However, when compared to the SCI + vehicle-treated animals, the SCI + NLX-112 group showed significant decreases in bladder weight and detrusor thickness (Figure 4B,C,E,F). In addition, the NLX-112-treated group showed clearly decreased developed edematous connective tissues (collagens were seen in blue color with Masson’s trichrome staining) compared to the vehicle-treated group (Figure 4B,C).

### 2.4. NLX-112 Treatment Significantly Decreases SCI-Upregulated Spinal 5-HT_1A_ Receptors

**Figure 5:**
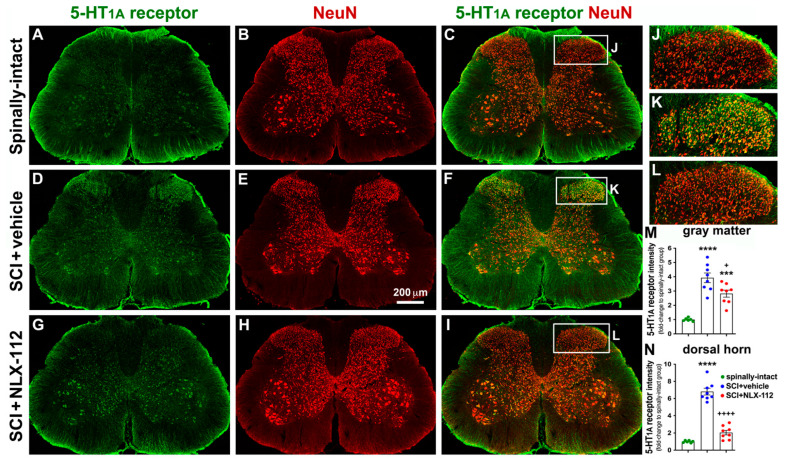
NLX-112 treatment significantly reduced SCI-increased 5-HT_1A_ receptors in the L4 spinal cord segment. Representative transverse sections with double immunostaining of 5-HT_1A_ receptor (green) and NeuN (red) are shown from spinally-intact (**A**–**C**), SCI + vehicle (**D**–**F**), and SCI + NLX-112 (**G**–**I**) animals. The higher magnification images (**J**–**L**) were taken from the dorsal horn region of the images in (**C**), (**F**), and (**I**), respectively. Statistical analyses of the intensity of 5-HT_1A_ receptors (mean ± SEM) in the gray matter (**M**) and dorsal horn (**N**) are shown among the groups. *** *p* < 0.001 and **** *p* < 0.0001 vs. spinally-intact group; + *p* < 0.05 and ++++ *p* < 0.0001 vs. SCI + vehicle group (one-way ANOVA with a Tukey’s post hoc test). N = 6 or 8 per group.

**Figure 6:**
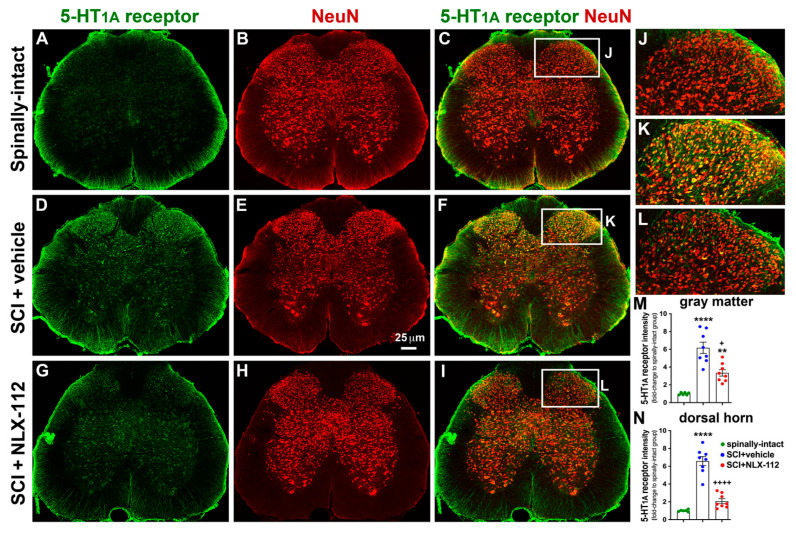
NLX-112 treatment significantly reduced 5-HT_1A_ receptors in the L6/S1 spinal cord segments after SCI. Representative transverse sections with double immunostaining of 5-HT_1A_ receptor (green) and NeuN (red) are shown from spinally-intact (**A**–**C**), SCI + vehicle l (**D**–**F**), and SCI + NLX-112 (**G**–**I**) animals. The higher magnification images (**J**–**L**) were taken from the dorsal horn region of the images in (**C**), (**F**), and (**I**), respectively. Statistical analyses of the intensity of 5-HT_1A_ receptors (mean ± SEM) in the gray matter (**M**) and dorsal horn (**N**) are shown among the groups. ** *p* < 0.01 and **** *p* < 0.0001 vs. spinally-intact group; + *p* < 0.05 and ++++ *p* < 0.0001 vs. SCI + vehicle group (one-way ANOVA with a Tukey’s post hoc test). N = 6 or 8 per group.

To further investigate the distribution of spinal 5-HT_1A_ receptors after contusive SCI and in response to NLX-112 treatment, an anatomical study was used to determine the location and amount of spinal 5-HT_1A_ receptors in the gray matter of the L4 (Figure 5) and L6/S1 (Figure 6) segments of the spinal cord. In the spinally-intact animals, the immunoreactivity of spinal 5-HT_1A_ receptors was relatively low in the gray matter of both the L4 and L6/S1 segments (Figure 5A and Figure 6A). The ventral horn appeared to have more immunoreactivity than the dorsal horn. However, significant upregulation of 5-HT_1A_ receptor expression was observed in the overall gray matter, especially in the dorsal horn, in the SCI + vehicle group while compared to the spinally-intact group in both the L4 and L6/S1 segments (Figure 5D,M,N and Figure 6D,M,N). Most 5-HT_1A_ receptors were expressed by neuronal cells (NeuN+) in both the L4 segment (Figure 5C,F,I) and the L6/S1 segment (Figure 6C,F,I) among all groups. In particular, significant upregulation of 5-HT_1A_ receptors was observed in the dorsal horn of the L4 segment (Figure 5K,N) and the L6/S1 segment (Figure 6K,N). The SCI + NLX-112 group also showed an overall increase in 5-HT_1A_ receptors in gray matter compared to the spinally-intact group in both the L4 (Figure 5G,M) and L6/S1 (Figure 6G,M) segments, but to a lesser extent than the SCI + vehicle group (Figure 5D,M and Figure 6D,M). Specifically, the SCI + NLX-112 group significantly decreased the amount of 5-HT_1A_ receptors in the dorsal horn, to a more obvious decrease than in the overall gray matter (Figure 5D,G and Figure 6D,G), while compared to the SCI + vehicle group in both the L4 (Figure 5D,G,M,N) and L6/S1 segments (Figure 6D,G,M,N).

## 3. Discussion

In the present study, we investigate the efficacy of long-term activation of spinal 5-HT_1A_ receptors by two-week delayed NLX-112 treatment after T8 contusive SCI. The main and novel findings of this study include that NLX-112 intervention can significantly (1) improve locomotion in a dose-dependent fashion, (2) improve LUT function (both detrusor and EUS activities), (3) reduce bladder weight and improve bladder morphology, and (4) reduce SCI-upregulated spinal 5-HT_1A_ receptor expression below the injured site in both the L4 and L6/S1 spinal cord segments.

The serotonergic system (serotonin and serotonergic receptors) plays an important role in modulating spinal circuit activity for locomotion control. Dysfunction in the serotonergic system can impair recovery of locomotion after SCI [16]. The long-term activation of 5-HT receptors by pharmacological intervention has demonstrated some efficacy in treating SCI [17]. Previous reports have shown that long-term treatment with the 5-HT_1A_/5-HT_7_ receptor agonist 8-OH-DPAT, or 5-HT_2_ receptor agonists (quipazine), or a combination of both 5-HT_1A_ receptor and 5-HT_2_ receptor agonists can improve locomotion and largely prevent the upregulation of spinal 5-HT_1A_ receptor densities caudal to complete SCI [12,13,14,18]. In the present study, we demonstrated improvements in locomotor recovery by long-term two-week delayed delivery of NLX-112 in contusive SCI rats. The potential mechanisms underlying the locomotion recovery with NLX-112 treatment are that NLX-112 may increase the excitability of the lumbar ventral motoneurons [19] and/or enhance the level of excitability of the motor network of the spinal cord (including central pattern generator), which in turn promote motor function [20]. This finding not only further supports the feasibility of pharmacological activation of 5-HT_1A_ receptors to improve locomotion but also advances the intervention to a delay for two weeks after SCI following a more clinically relevant contusive SCI instead of complete SCI.

Along with other parts of the autonomic nervous system, for example, the heart, vasculature, and airways [21], central 5-HT receptors have been implicated in the control of micturition [22,23,24]. These conclusions are based upon the use of pharmacological interventions with either agonists or antagonists to its receptors [25]. The 5-HT_1A_ receptor has been investigated in controlling micturition behavior in different species under normal physiological conditions [25]. The proof-of-concept of improving LUT function by acutely activating 5-HT_1A_ receptors with 8-OH-DPAT [4,5,6] or NLX-11 2 [15] after complete or contusive SCI has been established. In the present study, we investigate whether delayed and long-term activation of 5-HT_1A_ receptors by NLX-112 could improve LUT function (both detrusor and EUS activities) after T8 contusive SCI. Indeed, our findings that NLX-112 reduced detrusor overactivity and enhanced EUS bursting activity were consistent with the results of our previous acute pharmacological study [15]. Moreover, the improvements in bladder morphology, along with the reductions in bladder weight and the thickness of the bladder wall, supported the findings that NLX-12 treatment improves CMG and EUS EMG recordings. These data strongly suggest NLX-112 as the potential therapeutic strategy to treat neurogenic bladder after SCI.

Our recent study [26] and other reports [27,28,29] indicated upregulation of spinal 5-HT_1A_ receptors below the injured site after complete SCI. In the present study, upregulation of spinal 5-HT_1A_ receptors was observed following incomplete SCI (contusive model) in both the L4 and L6/S1 spinal cord segments. Particularly, more significant upregulation was observed in the dorsal horn following contusive SCI, which was consistent with what was found following complete SCI [26]. After SCI, serotonin levels below the site of injury, particularly in the dorsal horn, were significantly reduced [30]. Such reduction leads to hyperexcitability of dorsal horn sensory neurons due to the activation of 5-HT_1A_ receptors resulting in cellular hyperpolarization [31] and has been linked to mechanical allodynia and thermal hyperalgesia observed after SCI [11]. Therefore, SCI-mediated constitutive activation of 5-HT_1A_ receptors in the dorsal horn after SCI plays an important role in the hyperactivity of afferent inputs. Indeed, previous studies had shown that NLX-112 application could (1) induce analgesic effects in a formalin model of tonic nociceptive pain [32] and (2) induce c-Fos+ cells in the dorsal horn regions in the lumbar segment within 2–4 h, indicating its capacity to alter afferent inputs [33]. In addition, SCI can alter bladder afferent inputs to the spinal cord that can induce hyperactivity of the bladder involving unmyelinated, capsaicin-sensitive, C-fiber bladder afferent neurons [34]. Therefore, it is possible that NLX-112 reduces the SCI-upregulated 5-HT_1A_ receptors, leading to reduced hypersensitivity of sensory neurons from bladder afferent inputs. This observation also supports the reduction of non-voiding contractions in the CMG recordings with NLX-112 treatment after SCI. To conclude, this study has demonstrated the efficacy of a two-week delayed NLX-112 treatment in behavioral outcomes and beneficial effects in anatomical studies after T8 contusive SCI. From translational perspective, the current study and the presence of 5-HT_1A_ receptors in the human spinal cord and dorsal root ganglions [35,36] support that NLX-112 intervention is a potential repair strategy to treat SCI.

## 4. Materials and Methods

### 4.1. Animal Groups and Experimental Design

All animals were housed in standard laboratory cages under 12:12 h light–dark cycle conditions with standard rodent chow and water available ad libitum. All experiments were performed during the light cycle. All animal procedures were approved by the Cleveland Clinic Institutional Animal Care and Use Committee (protocol number 2239) and were performed in strict accordance with the recommendations in the Guide for the Care and Use of Laboratory Animals from the National Institutes of Health. All experimental procedures and data analysis were performed in a blinded fashion.

A total of fifty-two adult female Sprague-Dawley rats (220–250 g; Inotiv, Indianapolis, IN, USA) were used in this study, which included two protocols. In protocol 1, thirty animals were used to determine the optimal dose of NLX-112 (Neurolixis Inc.; Wilmington, DE, USA) using the BBB locomotion test. All animals received T8 contusive SCI. At two weeks post-SCI, all animals were randomized to five treatment groups (N = 6/group) to start treatment, including vehicle, NLX-112 (0.05 mg/kg), NLX-112 (0.1 mg/kg), NLX-112 (0.25 mg/kg), and NLX-112 (0.5 mg/kg). NLX-112, or vehicle treatment, was administered twice per day via intraperitoneal injection. The consideration of the dose selection, route and frequency of administration was based on the previous reports [33,37] and the recommendation from Neurolixis that provided NLX-112. All treatments started at 2 weeks post-SCI and then continued for an additional 6 weeks. The 2-week delay in intervention has significant advantages over an acute treatment protocol because not only (1) the bladder reflex does not return at least until 2 weeks post-SCI in rats, and (2) from a clinical perspective, the neurologic examination at 2 weeks post-injury is much more reliable than what done right after the injury. In protocol 2, the remaining twenty-two animals were used to determine anatomical outcomes and LUT function following treatment with the optimal dose (0.25 mg/kg) of NLX-112 obtained from protocol 1. The SCI model and the treatment methods, including the route of administration, starting date, and treatment duration, were the same as in protocol 1. There were three animal groups, including (1) the spinally-intact group (N = 6), (2) SCI + vehicle (N = 8), and (3) SCI + NLX-112 (0.25 mg/kg, N = 8).

The number of animals needed has been estimated based on our previous studies [15,26], which was largely dictated by the number of animals needed in each treatment group to obtain statistically significant results. Therefore, we used a sample size of 6 to 8 animals for each group for different measurements to achieve a power of 0.80 and a significance level of 0.05. We did not include data from any animal with premature death in the statistical analysis. Animals were only used for data analysis when they could complete all required procedures during the expected observation periods. Outliers were identified using the robust regression and outlier removal (ROUT) method with a Q of 1%. Functional assessments were compared using ANOVA. In this study, we used fifty-five rats, but three of them were excluded (one from protocol 1 and two from protocol 2) due to premature death or surgical complications after SCI.

### 4.2. T8 Contusive SCI Surgery

Rats were anesthetized by an intraperitoneal injection with ketamine (60 mg/kg) and xylazine (10 mg/kg). The paraspinal muscle layers were carefully dissected from T7–T9, and then a laminectomy was performed to expose the dorsal surface of the T8 spinal cord without damaging the dura. The vertebral column was stabilized by clamping the T7 and T9 vertebral bodies with two forceps fixed to the base of the Infinite Horizon Impact Device (IH-400, Precision Systems and Instrumentation, LLC, Lexington, KY, USA). The animals were placed on the platform, and the 2.5 mm diameter stainless steel impactor tip was positioned over the midpoint of T8, and then the spinal cord was impacted by 250 kDyne force. The overlying musculature was then sutured closed, and the skin was closed using 4-0 monofilament sutures (Ethicon USA, LLC, Somerville, NJ, USA). The animals were treated with buprenorphine and gentamycin for post-operative care. A force–displacement graph was used to monitor impact consistency. Any animal that exhibited an abnormal impact graph or greater than 10% deviation from 250 k Dyne force was immediately excluded from the study (n = 0). The bladder was manually expressed 2–3 times daily for two–three weeks until a voiding reflex returned.

### 4.3. The BBB Open Field Locomotion Test to Assess Hindlimb Locomotion

Two observers blinded to the treatment condition performed the locomotion test using the BBB locomotor rating scale. BBB scores were determined as described previously [38]. All SCI animals were assessed a week before the SCI procedures, the day after the SCI procedures, and then once per week after the SCI procedures until the end of the observation period.

### 4.4. Awake Urodynamics and EUS EMG Recording

Animals were acclimated to the universal restrainer 5 days prior to the awake urodynamics and EUS EMG recording. The first two days were 15 min each day. The third day was 30 min. The fourth day was 1 h. The fifth day was 3 h. Food treats were provided during acclimation. At the date of recordings, rats were anesthetized under 2.5–3% isoflurane with oxygen, followed by exposing the urinary bladder with an incision of the midline abdomen. A catheter (polyethylene 50) was inserted into the bladder through a small incision at the apex of the bladder dome. The bladder end of the catheter was secured with a purse-string suture on the opening of the detrusor. The end of the bladder catheter was connected to a pressure transducer (model P23XL-1; Gould Ohmeda, Valley View, OH, USA), an amplifier (Astro-Med, Inc., West Warwick, RI, USA), and a single-syringe infusion pump (World Precision Instruments, Sarasota, FL, USA) for signal recording and saline infusion. The bladder catheter exited from the rostral edge of the abdominal incision. The muscle layers and skin were also closed by 4-0 sutures. Two fine (50 μm diameter) Teflon-insulated platinum wire electrodes (A-M Systems, Carlsborg, WA, USA) were inserted percutaneously on both sides of the mid-urethra using a 27-gauge needle (Becton, Dickinson and Company, Franklin Lakes, NJ, USA) for subsequent measurements of EUS EMG activity. Next, rats were placed in a universal restrainer (Braintree Scientific, Inc., Braintree, MA, USA) for a 1 h recovery period from anesthesia prior to performing physiological tests. Two wire electrodes were connected to an AC amplifier (Astro-Med, Inc.) with high- and low-pass frequency filters at 10 Hz and 1 kHz and a recording system (DASH 8X, Astro-Med, Inc.) at a sampling frequency of 10 kHz. Continuous cystometrograms (CMGs) were collected using constant infusion (0.1 mL/min) of saline by an infusion pump through the catheter into the bladder to induce repetitive voids for 1 h to allow the LUT to adjust to the flow rate. After 1 h, the saline infusion was stopped, and the bladder was emptied by aspiration through the bladder catheter via a stopcock port. The pump was restarted, and after three to five reproducible voiding cycles, CMGs filled with EUS EMG recordings were obtained for further analysis, as described in our previous studies [15,26,39].

### 4.5. Quantification of CMG and EUS EMG Patterns

A MATLAB (The MathWorks, Inc., Natick, MA, USA) script was written to measure various CMG/EMG parameters. The segmented data included the full duration between the completion of two consecutive voiding contractions. CMG voiding parameters included voiding efficiency (%), peak voiding pressure (cm H_2_O), and bladder capacity (mL). The period of the voiding cycle from the empty bladder with saline infusion until the first micturition occurred was used to determine voiding efficacy. It was calculated by the percentage of voiding volume to the amount of total saline infused. The bladder capacity was measured by the first voiding volume following the saline infusion from the empty bladder. Non-voiding contractions (NVCs) were defined as contractions with amplitudes exceeding 5 cm H_2_O that were not associated with any successful micturition. In SCI animals, the series of NVCs preceding each voiding event were analyzed together to match their respective voiding events. The non-voiding parameters included the amplitude (cm H_2_O) and the number of non-voids during the bladder-filling period. The EUS EMG parameters included (during the bursting phase) the number of EMG bursts, EMG bursting duration (s), and EMG burst interval (s). EMG bursts were qualified as coordinated when they were timed in sync with the CMG high-frequency pressure oscillations (HFOs). Data are presented as mean ± standard error of the mean (SEM) for each group and were analyzed using a one-way ANOVA with a Tukey’s post hoc test using GraphPad Prism 9 (GraphPad, San Diego, CA, USA). The significance level was set to 0.05 for all comparisons.

### 4.6. Analyses of Bladder Morphology

The bladders of all animals were collected at the end of the observation period, and the wet weights were recorded. Bladders were further fixed with 4% paraformaldehyde (PFA) for 1 day and then transferred to 30% sucrose solution, sectioned transversely (10 μm) at the level of mid-bladder with a cryostat, and stained with Masson’s trichrome for additional morphological analysis under a light microscope. Ten random points were selected to determine the thickness of the bladder wall, and the distance between the lumen and the outside edge of the bladder wall around the cross-sectioned image was measured.

### 4.7. Immunohistochemistry for 5-HT_1A_ Receptors and Analyses of Immunoreactivity

At the end of the observation period, rats were terminated via transcardial perfusion with 4% PFA. The spinal cords were collected and stored in 4% PFA overnight and then transferred to a 30% sucrose solution. The lumbosacral segments, including L4 and L6/S1 segments, were sectioned at 30 µm with a cryostat for immunohistochemistry, and 15–20 sections (120 µm apart) from each animal were selected. The selected sections were placed in a blocking solution (PBS containing 0.5% Triton X-100 and 3% BSA) and then incubated with antibodies against 5-HT_1A_ receptors (MAB11041, Millipore Sigma, St. Louis, MO, USA) and NeuN (Abcam, Cambridge, UK) overnight at room temperature. All sections were washed three times with PBS for 10 min and then incubated at room temperature for 2 h with secondary antibodies (Abcam), including Alexa Fluor 488-conjugated secondary antibodies and Alexa Fluor 555-conjugated secondary antibodies. The sections were then washed three times with PBS, and mounting media (Vector Laboratory, Newark, CA, USA) was added before they were cover-slipped. The sections were photographed by a Zeiss confocal microscope for further analysis. The templates of standardized areas in the gray matter of L4 or L6/S1 segments were created for sampling in sections from each animal in each group. The mean number of pixels containing immunoreactive products in the sampled area (entire gray matter or dorsal horn only) was measured and multiplied by the average intensity. This value was subtracted from background immunolabeled intensity, as measured in a separate adjacent section. Graphs were plotted and statistics were assessed using GraphPad Prism 9.0 (GraphPad, Boston, MA, USA). The intensities are shown as mean ± SEM in units of the fold-change of the mean intensities from spinally intact rats, which are presented as 1. Significant differences between groups were determined (*p* < 0.05; *p* < 0.001) using an unpaired *t*-test. Light intensity and threshold values were maintained at constant levels for all analyses.

## Data Availability

Data are contained within the article.
